# Qualitative exploration of psychological reactions and coping strategies of breastfeeding mothers living with HIV in the Greater Accra Region of Ghana

**DOI:** 10.1186/s13006-017-0119-8

**Published:** 2017-06-24

**Authors:** Angela Kwartemaa Acheampong, Florence Naab, Adzo Kwashie

**Affiliations:** 1 School of Nursing, Wisconsin International University College-Ghana, Taifa-Burkina, P. O. Box TB 105, Accra, Ghana; 2 Department of Maternal and Child Health, School of Nursing, College of Health Science, University of Ghana, Legon, P. O. Box LG 43, Accra, Ghana; 3 Department of Education, Research & Administration, School of Nursing, College of Health Science, University of Ghana, Legon, P. O. Box LG 43, Accra, Ghana

**Keywords:** HIV and breastfeeding, Breastfeeding mothers, Psychological burden of mothers, Coping strategies

## Abstract

**Background:**

Exploring the psychological reactions of breastfeeding mothers living with Human Immunodeficiency Virus (HIV) is an important step which may improve guidelines for counselling. The purpose of this study was to explore the psychological reactions and coping strategies of breastfeeding mothers living with HIV in the Greater Accra Region of Ghana.

**Methods:**

Qualitative descriptive exploratory design was used to explore the psychological experiences and coping strategies of 13 breastfeeding mothers living with HIV in a main referral public hospital, at the greater Accra Region of Ghana. An interview guide was designed and piloted before it was used to collect data between November, 2014 and February, 2015. Data was content analyzed for themes and subthemes to emerge.

**Results:**

The two major themes that emerged included psychological reactions and coping strategies. Some of the subthemes were fear, anxiety, blame, hope, denial, prayer and trust in positive situations of life.

**Conclusion:**

The women used denial, prayer and hope in ART, among others to cope with their emotions. This highlights the need for HIV counsellors to detect signs of denial since it can lead to non-adherence to ART as well as relapse. Health workers should therefore put the coping strategies in context during counselling of mothers in this category.

## Background

The World Health Organization in 2010 recommended that mothers living with HIV should breastfeed for at least 1 year this is partly due to the compelling evidence about the benefits of breastfeeding to the HIV exposed infant [1]. Breastfeeding is one of the means through which HIV can be transmitted from mother to child and the rate of transmission is significantly reduced when the mother and her child are on antiretroviral therapy [1]. This may not negate the hyped, negative psychological emotions which mothers living with HIV attach to breastfeeding.

Breastfeeding is a cultural norm in Ghana. Among several communities in Africa, there is an inherent and indirect social pressure to breastfeed [2]. Consequently, all mothers are socially compelled to breastfeed irrespective of their HIV status. Once it is an established fact that there is a probability of transmitting the virus to the infant through breastfeeding, breastfeeding mothers living with HIV may experience certain psychological emotions. Mothers living with HIV feel a sense of responsibility for a possibility that the virus can be transmitted to their children through breastfeeding [3–5]. On the other hand, mothers living with HIV in countries where exclusive formula feeding is recommended for HIV exposed infants struggle with feelings of guilt and blame regarding not breastfeeding [6]. Mothers who transfer the virus to their children live with guilt for the rest of their lives [7]. There is also a feeling of shame and worthlessness among mothers who transmit the virus to their children [8].

People living with HIV in Ghana are stigmatized [9–11]. In a Ghanaian study, it was found that, stigmatization of people living with HIV occurred not only in the community but also at the hospital [12]. Although stigmatization in Ghana is experienced by the majority of the people living with HIV, it is much more intense towards women and men who have sex with men [9, 11]. The stigma is extended to some pediatric caregivers in Ghana [13]. In a study in Cameroon, half of the people living with HIV felt ashamed of themselves [14]. Stigma among women living with HIV generally increases from pregnancy to the postpartum period [15]. Individuals living with the virus are depressed due to gross discrimination [16]. This is due to the fact that, the mode of transmission of the virus is deemed unclean and evil by most Africans, and therefore, most of them are discriminated against [17]. Psychologically, the depression and anxiety levels of people living with the virus are higher than those in the normal population [8]. Women who report greater stigma are more likely to have increased symptoms of anxiety [15] and that may aggravate concerns about vertical transmission through breastfeeding. Prenatal depression among women living with HIV has a negative impact on exclusive breastfeeding [18]. Mothers with postpartum depression have more than six times probability of not practicing exclusive breastfeeding and four times increased chance of having children who are underweight [19]. Levy and colleagues (2010) showed through their analysis that psychological disturbances led to physiological consequences and the disease progression from HIV infection to AIDS was rapid, when the people living with the virus suffer psychological distress [20].

Few studies have explored the psychological reaction and coping strategies of breastfeeding mothers living with HIV in Africa. This part of the world that is home to over 60 % of the world’s HIV/AIDS population. This study explored the psychological reactions and coping strategies of breastfeeding mothers living with HIV in the Greater Accra Region of Ghana.

## Methods

A Qualitative descriptive exploratory design was used to explore the psychological reactions and coping strategies of 13 breastfeeding mothers living with HIV in a main referral public hospital in the Greater Accra Region of Ghana. The period for data collection was between November 2014 and February 2015. A semi-structured interview guide with open ended and probing questions was used to collect data from participants who had been purposely selected to be involved in the study. Participants were asked to express how they felt about their decision to breastfeed. The criteria for inclusion in the study included voluntary participation, being a breastfeeding mother living with HIV in addition to receiving anti-retro viral therapy and the ability to communicate fluently in either ‘Twi’ (local dialect) or English. Eligible mothers receiving anti-retroviral therapy (ART) were approached on clinic days after permission had been granted by the gatekeepers of the hospital and the objective of the study was explained to them. Those who voluntarily opted to participate in the study were enrolled. The interviews were conducted by the principal investigator in a quiet consulting room at the ART unit of the hospital. Only one participant at a time was interviewed by the researcher thereby ensuring privacy. The women were asked to express their feelings about their decision to breastfeed. Measures put in place to ensure methodological rigor included protracted interaction with participants, member checking of equivocal responses, triangulation of data and persistent observation by the researcher. For anonymity, pseudonyms and participants’ age were used to differentiate between participants. Before the women were purposefully sampled to participate in the study, ethical clearance was sought from the Institutional Review Board of the Noguchi Memorial Institute for Medical Research at the University of Ghana with ethics number NMIMR-IRB CPN 005/14-15. The participants were given information sheets and consent forms to sign before data was collected from them. Each interview was audio-taped with participants’ permission and transcribed verbatim. Content analysis was used to analyze data as described by Padgett [21]. Data was managed manually. Colors were used to differentiate between themes and subthemes. Data were first coded by attaching words or phrases that captured the essence of each datum. After coding, similar codes were grouped together to form subthemes and then collated to form major themes.

## Results

The two themes that emerged after content analysis were: psychological reaction and coping strategies. Seven subthemes emerged. The feelings expressed among the participants in this study included fear, anxiety and blame. They used hope, denial, prayer, trust in positive situations of life and ART to cope with their psychological reactions.

### Demographic characteristics

The ages of the participants ranged between 29 and 40 years. All the 13 participants had more than one child. All the participants were married. Two of the participants were unemployed and the rest worked in the informal sector.

### Psychological reactions

All the 13 participants shared both positive and negative experiences about their psychological reactions. Some of the participants had already dealt with the psychological burden of breastfeeding with HIV and were leading normal lives that had a more positive outlook which made them cope better than the others who had not overcome their feelings of fear, blame and anxiety.

#### Fear

Fear was an unpleasant psychological emotion which was expressed by the participants as a result of the perceived impending threat of transmitting the HIV to their infants through breastfeeding. The inconveniences involved in living with a chronic infection such as HIV may have been the possible reason why most mothers were afraid of transmitting the virus to their infants. Seven participants were still afraid of transmitting the virus to their children through breastfeeding. Women expressed fears and stated:

*“fear of transmission to my child after breastfeeding is still there. This is because at birth, when the test is done, the child might be HIV negative but later, after breastfeeding for a while, the child may come out as HIV positive” (Aba, 29 years). “I am afraid from time to time concerning the possibility of transmitting the virus to the child through breast milk…” (Kakraba, 32 years).*

#### Anxiety

The feeling of nervousness about the uncertain HIV statuses after their children had been breastfed was voiced in the form of anxiety by the women. Five participants were anxious about the eventual outcome of their children after the last confirmatory test at 18 months. They kept on wondering what the results of the HIV test was going to be after the period of breastfeeding since most of the mothers were aware of the fact that breast milk was one of the modes of transmission for HIV from mother to child. Two of the anxious women expressed their anxiety as described below:

*“I think about it from time to time. I sometimes ask myself certain questions like; what if this breast milk I am giving to this child predisposes her to the HIV? If the child gets the HIV through the breastfeeding, what would I do? (Kukua, 37 years). “Sometimes, I think about it that; ehh what if at the beginning, although the test said HIV negative, what if at one and a half years we go back and do the final test and it becomes HIV positive? (Adwo, 32 years)*

#### Blame

The women felt a sense of taking responsibilities for the consequences of their actions by blaming themselves. Eight participants blamed themselves for being HIV positive and felt that, if their children contracted the virus through breastfeeding from them, it would solely be their fault.

*“... I blame myself that if the child gets the HIV, it would surely be my fault. Therefore, I always blame myself first. Especially when I got to know that my husband was negative, I give all the blame to myself...” (Baaba, 34 years)“…but I blame myself all the time….” (Aba, 29 years)*

On the contrary, one participant had an opposing view on the issue of blame. She was of the view that, the mother cannot be blamed even if the child contracts the HIV through breastfeeding since the mother may not even know the origin of the infection. She expressed her views about blame as:

*“As for blame, I don’t think anyone should blame herself if the child becomes HIV positive through breastfeeding because, we don’t know where this disease is coming from….” (Araba, 35 years).*

### Coping strategies

#### Hope

Four of the women had overcome their negative psychological burdens and they were very hopeful since they led normal lives. Hope is a positive emotion that can improve the psychological outcomes of such mothers. One of them was of the opinion that, once her baby looked healthy physically, there was nothing to worry about she had the following to say:

*“I have hope. This is because of the fact that, by looking at my son, I have no doubt that he will breastfeed successfully and will be HIV negative. He looks so well although he is being breastfed…” (Baaba, 34 years).*

Some of the participants superstitiously believed that, since they sinned in the form of fornication to contract the virus, nature will make it in such a way that, their children would be spared and that was where their hopes emanated from. To those participants, children are considered blameless and sinless, therefore, they are not supposed to suffer from such an infection. One participant referred to HIV infection as a punishment for those who had sinned and acknowledged that:

*“no, no, no, no. I’m not afraid and I have faith and hope. I believe that if one has faith, nothing bad can happen. I believe that I am the one who has sinned and the child is innocent so if there should be any punishment, I should be punished with the disease and not the innocent child. So if I am the one who has sinned, I should carry my own cross”* (*Efua, 32 years*).

#### Denial

Six of the participants had found ways to push their HIV positive status away in their minds so that it was always viewed by them as a distant memory. One of the participants had also dealt with her psychological burden by denying the fact that the infection is a chronic disease which lives with the individual for life. She had referred to the HIV infection as a passing “wind” which would disappear in no time. She reported that:

*“This HIV can be a wind. I heard that one can contract it though urination, the surgery that was done for me, from food or even the salon. My husband is negative. It is only myself and my child who are HIV positive. So it is even possible that I had it through the pregnancy. Then I told my husband that, every disease is like the wind and that this one too shall pass” (Asaabea 39 years).*

According to the women, denying their HIV positive status helped them to breastfeed without any anxiety or guilt. Some women reported that, since they were told of their HIV positive status, they have purposefully pushed such information out of their minds and pretended to be HIV negative. With denial, they assumed that they were HIV negative and therefore had no possibility of transmitting the virus to their children through breastfeeding. One woman had the following to say:

*“I don’t believe that I am HIV positive that is why I am able to cope with the psychological burden” (Esaaba, 40 years).*

#### Prayer

Twelve participants firmly believed the act of praying would stop the HIV transmission to their children through breastfeeding. They attributed their confidence to the fact that they had a supreme being who was intervening on their behalf. Prayer ran through all of their shared experiences no matter their religious backgrounds. One participant’s prayer was directed in such a way that, God would spare her children from HIV infection even though she was breastfeeding. She had this to say:

*“Prayer is one of my coping strategies. Anytime I pray, I tend to tell God that, my children should always come out HIV negative. …” (Efe, 30 years). “… when I start having psychological distress, I start praying immediately… I tell God to miraculously prevent my children from getting infected with the virus through me” (Aba, 29 years).*

One of the women was skeptical about the issue of miracles through prayer in relation to HIV infection. She was of that view since she had seen a lot of people who had been deceived by pastors, and told that they had been healed so they should stop taking their medication, only to return later in a worst state since the HIV infection would have progressed to the disease stage (AIDS). She was doubtful due to her previous negative experience about miracles and prayer. She narrated the following about prayer:

*“Oh as for prayers, we all go for prayers. But in going for prayers, one must be vigilant, because a fake pastor can tell you that he has miraculously healed you and the virus is no longer in you. Such people who are tricked mostly decide not to come for their medication. But they are always brought back to the hospital worse than before.” (Araba, 35 years).*

#### Trust in the positive situations of life

This section describes how some of the women had lifted themselves up personally to look beyond their circumstances and focus on other positive situations in their lives. Some of the women had accepted the fact that, they were the best persons to encourage themselves since they were the ones experiencing the psychological burden. Five of the women had encouraged themselves and were ready to face any form of circumstance ahead at any time. Their sense of optimism was so high that, they were comfortable with their lives psychologically. One woman had this to share:

*“If I allow it to be my burden I will always panic and brood over the fact that this is the disease that would kill me. Now if I panic and eventually die, who will take care of my children? I have to be strong so that I can grow old in order to see my children grow. So if I think about my status and die right now, if my children grow older, who will tell them about this reality” (Efua, 32 years).*

#### Hope in ART

The presence of effective anti-retroviral medication has made it possible for people living with HIV and their children, a chance to live normal lives. Another way through which participants coped with their psychological burdens was the hope they had in ART. According to these women, the effect of the medication reduced the chances of transmission of the virus from mother to child through breastfeeding. Ten of the participants were optimistic about their circumstances because of the availability of antiretroviral medication for themselves and their children whom they were breastfeeding. The women indicated that, the anti-retroviral medication has given them a new sense of hope and it has reduced the probability of the virus being transmitted from mother to child through breastfeeding.

*“As for me, my ability to cope is because of the fact that I am on ART. The moment it is 8:30, the father of this child reminds me by saying “hei, are you not going to give the animals in you food?” Then I would also ask him “which animals?” then he will respond jovially “the animals in your stomach”. Then we would all laugh jovially about it” (Esaaba, 40 years). “I also believe that, once the mother is on anti-retroviral therapy, even if the child does not receive ART at birth and the mother believes herself, the child might not get the virus from the mother” (Araba, 35 years).*

## Discussion

There is an array of emotions that linger in the minds and hearts of breastfeeding mothers living with HIV. Some are emotions that promote breastfeeding. On the other hand, others are negative and may suppress the flow of milk and dampen the spirit of the breastfeeding mothers. The women reported fear, anxiety and blame.

Although the chances of transmitting the HIV to infants through breastfeeding reduces drastically when a mother is given anti-retroviral prophylaxis, [22–27] the women expressed fear. The findings are similar to that of studies conducted by Kanniappan et al. (2008) in India, Visser et al. (2008) in South Africa and Levy et al. (2010) in Malawi [20, 28, 29]. The implications of living with a chronic infection such as HIV may have been the possible reason why most mothers were afraid of transmitting the virus to their infants. Contracting the virus means that, the child would have to be given anti-retroviral prophylaxis for a period of time before a possible cure is found to treat the infection in the future. This would also predispose their children to stigmatization [17]. In a conservative culture such as Ghana, people living with HIV are stigmatized in the community and sometimes in the hospitals [9–11]. Perhaps, the possibility of side effects after prolonged exposure of their children to the anti-retroviral medication if they contract HIV, may also be causing the fear in the mothers. The above mentioned repercussions of stigma and possible side effects of antiretroviral therapy may aggravate the anxiety of mothers living with HIV with regards to transmission through breastfeeding.

Anxiety was also mentioned as one of the emotions the women experienced. The women were uncertain about the future prediction of their children’s HIV status. They expressed this emotion through questioning the act of breastfeeding. Anxiety in women living with HIV has been reported in some studies [20, 30] which is consistent with the current findings. The anxiety may be heightened in the women because of the waiting period involved in confirming the HIV statuses of their infants. The infants go through a series of tests from birth to 18 months of age when the final confirmatory test is done. So it is only at 18 months when the mother can ascertain the HIV status of her infant. A study conducted in the United States found that, mothers went through trauma and stress during each HIV test until the result of the test was released [31]. This therefore has culminated in the state of anxiety in the women.

The infant is widely considered by the Ghanaian society as blameless. Therefore, any mishap that occurs to infants as a result of transmission through body fluids from mother to child is vehemently scrutinized, with all hands pointing at the mother. Blame was a deep seated feeling that emanated from any thoughts of transmitting the virus through breastfeeding, and flashed through the women’s minds. Perhaps blame left the women wondering how their children’s future would eventually unfold. This contemporary finding is like that of other studies whereby the mothers always blamed themselves for being responsible for whatever their infants were going through [3–5]. Contrary to such views, one woman had a different perspective so far as blame was the subject matter for discussion. She was of the view that, the mother can never be held responsible if the child contracted the virus through breastfeeding. It could be that, the woman had overcome her psychological burden of blame and was leading a more positive lifestyle. The blame could be as a result of the fact that, the rate of HIV transmission through accidental means is minimal. A lot of the women may have felt that, having unprotected sex may have predisposed them to the virus thereby making them feel responsible for their HIV positive status. The women may have concluded that, the act of engaging in unprotected sex that led to contraction of the virus could have been avoided. HIV counselors must place emphasis on the psychological reactions of clients and focus on activities that can reduce the burden of blame on breastfeeding mothers living with HIV.

The women had to find different ways to mitigate the psychological reactions they encountered on daily basis. Among the coping strategies adopted by the women to counteract the emotions were hope, denial, prayer, trust in the positive situations of life and hope in ART.

In the midst of all the negative psychological reaction to the act of breastfeeding in the context of HIV is hope. Some of the women had a glimmer of hope that at the end of the 1 year breastfeeding period, their children would be uninfected with HIV. This is similar to a study by Sanders [4]. On the contrary, other studies reported fear among such mothers [20, 30]. A possible reason for the mothers’ optimism could be the improvement in the quality of anti-retroviral medication which has turned HIV infection from a death sentence in the past into a chronic infection which can be managed for the individual to lead a normal lifestyle. Better improvement in the medication regimen which may possibly change the daily medication intake into one tablet per week, can further normalize the lives of people living with HIV especially the breastfeeding mothers and give them hope to face motherhood.

Denial is the brain’s way of dealing with stress which involves mentally refusing to accept the truth [32]. This mechanism has both advantages and disadvantages. The advantage could be that, it helps individuals to deal with stress in a positive manner. On the other hand, it may lead to a situation whereby an individual accepts the truth as false and will therefore refuse to get help. To them, denial helps in alleviating the psychological distress which they face on daily basis. Denying their HIV status was a way of dealing with the news in such a way that, they could lead normal lives like any ordinary mother in the community. The women reported that, the long waiting period of 18 months to confirm the HIV status of their children increases their psychological burden as they breastfeed. Therefore, one of the ways to ignore the brain’s response to such stress is to deny the existence of HIV in their blood or body fluids like breast milk. Every mother wants to have a normal healthy child without any blemish. Therefore, the thought of possibly transmitting a deadly virus to a child through breast milk may be thought provoking and stressful.

Prayer is a means of communicating to God in both the spiritual and physical realms. Prayer as a coping strategy was adopted by all the women with the exception of one. To the majority of the women, they serve a supreme being who does not forsake them. That supreme God is the one that consoles them through the words in either the Bible or Quran. Therefore, their thoughts, worries and feelings were channeled towards this God who was to them, mightier than anyone on earth. Such proclamations allowed the women to pour out their hearts’ desires, which is healing in itself. The women were therefore optimistic that, once they had channeled their worries to their God through prayer, their God would console them. The typical Ghanaian society is made up of people who believe in the spirit of God and His existence. Most of them worship God with the hope that, all their problems would be solved eventually.

On the other hand, modernization and acculturation may have influenced the one woman who believed in practicality rather than supernatural beings. To her, believing in God alone was not enough to solve her problems. She was more practical and open to the fact that, praying and not taking medication would cause disease or infection relapse, which may put the lives of the mother and child in jeopardy. Therefore, she had more faith in the medication than prayer. Due to the influence of culture on the lives of individuals, counselors must always make it a priority to direct their clients to cope with psychological burdens by believing in the God they serve. On the other hand, counselors must conscientize the mothers not to neglect the physical aspect which involves the intake of anti-retroviral medication to keep the viral load at controllable levels.

Some women had resolved to trust in positive situations of life as a self-motivating technique, notwithstanding the fact that there was a chance of transmitting the virus through breast milk. The above finding about trust in positive situations of life is consistent with findings from different parts of the globe [33–40]. The women felt that, once they had given birth, burdening themselves psychologically may lead to an increased deterioration in the disease. Meanwhile, every mother would like to see her children grow, so a sense of survival was key to realizing that dream. Therefore, they ensured that, their line of negative thoughts was reversed. Perhaps, a child brought some normalcy to the lives of these women. Some years ago, living with HIV and having normal healthy children was impossible. Self-motivation can therefore be adopted to restore confidence in the lives of mothers living with HIV.

The use of anti-retroviral therapy was also mentioned as a coping strategy by the women. Similar findings were reported in a study that, the mothers attributed their confidence and sense of hope to the presence of the anti-retroviral therapy [41]. The women may have listed the presence of anti-retroviral therapy as a coping strategy because of the fact that, women who took the medication during breastfeeding mostly had their children emerging as HIV negative. The women kept referring to other women on anti-retroviral therapy who had breastfed successfully, for their children to also become HIV negative. It could also be attributed to the previous experience of the women. Most of the women with positive previous experience of breastfeeding and anti-retroviral medication were still hopeful that the current children would similarly breastfeed and become HIV negative if they continue taking the medication.

## Conclusion

Some of the participants had overcome their psychological burdens to an extent that they were encouraging other mothers in similar situations to breastfeed with hope. Some of the breastfeeding mothers living with HIV were still sad about their situation because of the anxiety that is associated with the uncertainty of transfer of the virus from mother to child. Coping strategies such as denial, prayer and hope in ART were used to overcome some of the negative psychological reactions. The findings of this study would inform policy makers on the psychological reactions of breastfeeding mothers living with HIV in Ghana. The findings may be used to design counselling tools specifically for breastfeeding mothers living with HIV in Ghana. Nurses and midwives who have been specially trained to counsel breastfeeding mothers living with HIV can design a specific intervention tool which would identify feelings of fear, anxiety and blame among such mothers. The tool should also have guidelines that would guide HIV counsellors to assist breastfeeding mothers to overcome such negative feelings.
